# kruX: matrix-based non-parametric eQTL discovery

**DOI:** 10.1186/1471-2105-15-11

**Published:** 2014-01-14

**Authors:** Jianlong Qi, Hassan Foroughi Asl, Johan Björkegren, Tom Michoel

**Affiliations:** 1 School of Life Sciences – LifeNet, Freiburg Institute for Advanced Studies (FRIAS), University of Freiburg, Freiburg, Germany; 2 Epigenomic Mapping Centre, McGill University, Montreal, Canada; 3 Cardiovascular Genomics Group, Division of Vascular Biology, Department of Medical Biochemistry and Biophysics, Karolinska Institute, Stockholm, Sweden; 4 Department of Medical Pathology and Forensic Medicine, University of Tartu, Tartu, Estonia; 5 Division of Genetics & Genomics, The Roslin Institute, The University of Edinburgh, EH25 9RG Easter Bush, Midlothian, UK

**Keywords:** eQTL, Non-parametric methods, Matrix algebra, Software

## Abstract

**Background:**

The Kruskal-Wallis test is a popular non-parametric statistical test for identifying expression quantitative trait loci (eQTLs) from genome-wide data due to its robustness against variations in the underlying genetic model and expression trait distribution, but testing billions of marker-trait combinations one-by-one can become computationally prohibitive.

**Results:**

We developed kruX, an algorithm implemented in Matlab, Python and R that uses matrix multiplications to simultaneously calculate the Kruskal-Wallis test statistic for several millions of marker-trait combinations at once. KruX is more than ten thousand times faster than computing associations one-by-one on a typical human dataset. We used kruX and a dataset of more than 500k SNPs and 20k expression traits measured in 102 human blood samples to compare eQTLs detected by the Kruskal-Wallis test to eQTLs detected by the parametric ANOVA and linear model methods. We found that the Kruskal-Wallis test is more robust against data outliers and heterogeneous genotype group sizes and detects a higher proportion of non-linear associations, but is more conservative for calling additive linear associations.

**Conclusion:**

kruX enables the use of robust non-parametric methods for massive eQTL mapping without the need for a high-performance computing infrastructure and is freely available from http://krux.googlecode.com.

## Background

Genome-wide association studies have identified hundreds of DNA variants associated to complex traits including disease in human alone [1]. To understand how these variants affect disease risk, genotype and organismal phenotype data are integrated with intermediate molecular phenotypes to reconstruct disease networks [2]. A first step in this procedure is to identify DNA variants that underpin variations in expression levels (eQTLs) of transcripts [3], proteins [4] or metabolites [5]. As modern technologies routinely produce genotype and expression data for a million or more single-nucleotide polymorphisms (SNPs) and ten-thousands of molecular abundance traits in a single experiment, often repeated across multiple cell or tissue types, the number of statistical tests to be performed when testing each SNP for association to each trait is huge. Furthermore, multiple testing correction requires all tests to be repeated several times on permuted data to generate an empirical null distribution. Despite being trivially parallelisable, the computational burden of testing SNP-trait associations one-by-one quickly becomes prohibitive.

Recently a new approach ("matrix-eQTL") was developed which uses the fact that the test statistics for the additive linear regression and ANOVA models can be expressed as multiplications between rescaled genotype and expression data matrices, thereby realising a dramatic speed-up compared to traditional QTL-mapping algorithms [6]. A limitation of these models is their assumption that the expression data is always normally distributed within each genotype group. For this reason, QTL and eQTL studies have frequently used non-parametric methods which are more robust against variations in the underlying genetic model and trait distribution [7,8]. In particular, the non-parametric Kruskal-Wallis one-way analysis of variance [9] does not assume normal distributions and reports small *P*-values if the median of at least one genotype group is significantly different from the others [8].

Here we report a matrix-based algorithm ("kruX"), implemented in Matlab, Python and R, to simultaneously calculate the Kruskal-Wallis test statistics for several millions of SNP-trait pairs at once that is more than ten thousand times faster than calculating them one-by-one on a human test dataset with more than 500,000 SNPs and 20,000 expression traits. Additional benefits of kruX include the explicit handling of missing values in both genotype and expression data and the support of genetic markers with any number of alleles, including variable allele numbers within a single dataset.

## Implementation

### Input data

KruX takes as input genotype values of *M* genetic markers and expression levels of *N* transcripts, proteins or metabolites in *K* individuals, organised in an *M* × *K* genotype matrix **G** and *N* × *K* expression data matrix **D**. Genetic markers take values 0,1,…,*ℓ*, where *ℓ* is the maximum number of alleles (*ℓ* = 2 for biallelic markers), while molecular traits take continuous values. We use built-in functions of Matlab, Python and R to convert the expression data matrix **D** to a matrix **R** of data ranks, ranked independently over each row (i.e. molecular trait). KruX assumes that the input expression data has been adjusted for covariates if it is necessary to do so [10,11] and all data quality control has been performed.

### Calculation of the Kruskal-Wallis test statistic by matrix multiplication

The genotype matrix **G** is first converted to sparse logical index matrices **I**_*i*_ of the same size, where **I**_*i*_(*m*,*k*) = 1 if **G**(*m*,*k*) = *i* and 0 otherwise (*i* = 0,…,*ℓ*). Next observe that the 1 × *M* vector **N**_*i*_ with entries $N_{i}(m)=\sum_{k=1}^{K}I_{i}(m,k)$ and *N* × *M* matrices **S**_*i*_ with entries

(1) $$S_{i}(n,m)=\overset{K}{\underset{k=1}{\sum }}R(n,k)I_{i}(m,k)=\left(R\cdot I_{i}^{T}\right)(n,m),$$

are respectively the number of individuals and the sum of ranks for the *n*th trait in the *i*th genotype group of the *m*th marker. We can then calculate an *N* × *M* matrix **S** with entries

(2) $$S(n,m)=\frac{12}{K(K+1)}\overset{\ell }{\underset{i=0}{\sum }}\frac{S_{i}{\left(n,m\right)}^{2}}{N_{i}(m)}-3(K+1),$$

using efficient vectorised operations. If none of the rows in **D** contain ties, then each entry **S**(*n*,*m*) equals the Kruskal-Wallis test statistic for testing trait *n* against marker *m*[9]. For markers with less than the maximum of *ℓ* genotype values, 0/0 division will result in NaN columns in the intermediate matrices with entries **S**_*i*_(*n*,*m*)^2^/**N**_*i*_(*m*) for the empty genotype groups. By replacing all NaN’s by zeros before making the sum in eq. (2), the corresponding entries in **S** will be the correct statistics for a test with fewer than *ℓ* degrees of freedom. Thus we need *ℓ* + 1 matrix multiplications and the associated element-wise operations to calculate the test statistic values for all marker-trait combinations.

### P-value calculation and empirical FDR correction

KruX takes as input a *P*-value threshold *P*_*c*_, calculates the corresponding test statistic thresholds for *d* degrees of freedom (*d* = 1,…,*ℓ* - 1), and identifies the entries in *S* which exceed the appropriate threshold value. For these entries only a *P*-value is calculated using the *χ*^2^ distribution. Empirical false-discovery rate (FDR) values are computed by repeating the *P*-value calculation (with the same *P*_*c*_) multiple times on data where the columns of the expression data ranks are randomly permuted. The FDR value for any value *P* ≤ *P*_*c*_ is defined as the ratio of the average number of associations with *P*^′^ ≤ *P* in the randomised data to the number of associations with *P*^′^ ≤ *P* in the real data.

### Handling missing values

When data values are missing for some marker or trait, all test statistics for that marker or trait need to be adjusted for a smaller number of observations. For the expression data, missing values are easily handled since the ranking algorithms will give NaN’s the highest rank. By setting the entries corresponding to missing values in **D** to zero in **R**, eq. (1) still produces the correct sums of ranks, while the matrix multiplication

$$\begin{array}{l} \left(Z\cdot I_{i}^{T}\right)(n,m)=\overset{K}{\underset{k=1}{\sum }}Z(n,k)I_{i}(m,k)=N_{i}(n,m), \end{array}$$

where **Z** is the *N* × *K* matrix with **Z**(*n*,*k*) = 0 whenever **D**(*n*,*k*) = NaN and 1 otherwise, produces the corrected number of individuals in the *i*th group of the *m*th marker for the *n*th trait. Replacing the constant *K* in eq. (2) by a *N* × *M* matrix **K** where **K**(*n*,*m*) is the number of non-missing samples for trait *n* and performing element-wise division and substraction operations then gives the correct test statistic for all pairs.

Handling missing genotype data is less easy because the expression ranks that need to be adjusted are specific to each marker-trait combination (e.g if a marker has a missing value where a trait has rank *r*_1_, then all samples with ranks *r* = *r*_1_ + 1,…,*K* need to be lowered by 1). KruX uses the fact that missing genotype values are generally due to sample quality and therefore patterns of missing values are often repeated among markers. For each unique missing value pattern, a new genotype matrix for all markers with that pattern and a new expression data matrix with the corresponding samples removed are constructed to calculate the test statistics for all affected marker gene combinations. Missing genotype data increases the computational cost of the algorithm considerably and it is recommended to limit the number of missing values by only considering markers with a sufficiently high call rate.

### Handling tied data

In the presence of tied observations, the statistic in eq. (2) needs to be divided by a factor $1-\frac{\sum T}{K^{3}-K}$, where the summation is over all groups of ties and *T* = *t*^3^ - *t* for each group of ties, with *t* the number of tied data in the group [9]. The factor *T* is automatically computed for each trait during the ranking step and the matrix *S* is therefore easily corrected using element-wise matrix operations (Matlab version only). Whereas ties are usually rare in standard gene expression datasets, the ability to handle tied data expands the scope of kruX to count-based, discretised or qualitative data types.

### Data slicing

Since kruX needs to create intermediate matrices of size *N* × *M*, where *N* is the number of traits and *M* the number of markers, which do not usually fit into memory for large datasets, kruX supports the use of data ‘slices’ to divide the complete data into manageable chunks. In typical applications, the number of markers is one or two orders of magnitude larger than the number of traits. Therefore the default behaviour of kruX is to keep the expression data as a single matrix and simultaneously test all traits against subsets of markers. The user can provide either a slice size and kruX will process marker blocks of this size serially, or a slice size and initial marker and kruX will process a single slice starting from that marker. The latter option allows trivial parallelisation across multiple processors.

## Results and discussion

### Validation data

To test kruX we provide example analysis scripts and a small anonymised dataset of 2,000 randomly selected genes and markers from 100 randomly selected yeast segregants [12]. Here we describe an application of kruX on a human dataset of 19,610 genes and 530,222 SNP markers measured in 102 whole blood samples from the Stockholm Atherosclerosis Gene Expression (STAGE) study [13]. All SNPs in the dataset had minor allele frequency greater than 5%, no missing values and probability to be in Hardy-Weinberg equilibrium greater than 10^-6^.

### kruX is exact and fast

**Figure 1:**
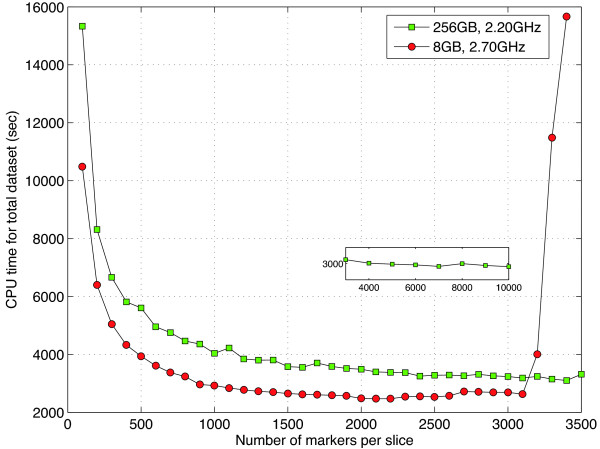
**kruX runtime on STAGE data.** Total extrapolated single-CPU run time in seconds for the Matlab implementation of kruX for different numbers of SNP markers per data slice (see main text for details). Green squares are times on a high-memory server with 256 GB RAM and 2.20 GHz processor and red circles are times on a laptop with 8 GB RAM and 2.70 GHz processor. The insert shows the continuation of the green squares upto a slice size of 10,000 markers.

We first confirmed that kruX produces the same results as testing marker-trait combinations one-by-one using the built-in Kruskal-Wallis functions to verify the correctness of our implementations. To test the performance of kruX we divided the genotype data into slices of variable size and extrapolated the total run time from running a single genotype data slice against all expression traits and multiplying by the number of slices needed to cover the entire set of 530,222 SNPs. The total run time rapidly decreases until a genotype slice contains about 1,000 SNPs and stays almost constant thereafter. On a laptop with 8 GB RAM, the limit is reached at around 3,000 SNPs per slice after which run time sharply increases again due to memory limitations (Figure 1). We therefore recommend a genotype slice size of around 2,000 markers, resulting for this dataset in around 250 separate jobs, which will take around 2,500 seconds (42 minutes) when run serially on a single processor. By comparison, the total extrapolated run time when computing all 19,610 × 530,222 associations one-by-one using the built-in Kruskal-Wallis function on the same hardware as in Figure 1 are respectively 4.8 · 10^7^ (256 GB, 2.20 GHz server) and 2.6 · 10^7^ (8 GB, 2.70 GHz laptop) seconds such that kruX is respectively 17,000 and 11,000 times faster on this particular dataset. On the same dataset and hardware, the comparatively simpler matrix operations for the parametric tests in matrix-eQTL took respectively 5 minutes (linear model) and 7.4 minutes (ANOVA model).

### The Kruskal-Wallis test is more conservative than corresponding parametric tests

**Figure 2:**
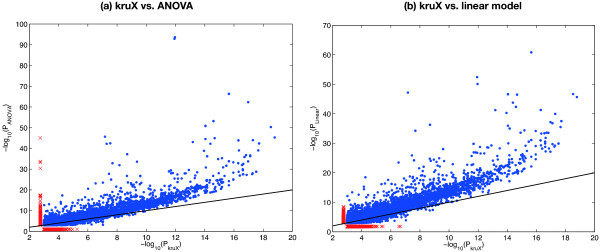
**Comparison of kruX vs. parametric ANOVA and linear models.** Comparison of nominal non-parametric *P*-values calculated by kruX vs. parametric ANOVA **(a)** and linear models **(b)**, showing all *cis*-acting eQTL-gene pairs with *P* < 10^-3^ detected by both methods (blue dots) and by only one of the methods (red crosses). The black line indicates the line with slope *y* = *x*.

**Figure 3:**
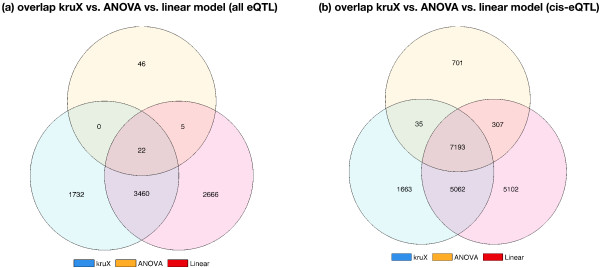
**Comparison of kruX vs. parametric ANOVA and linear models.** Comparison of all eQTL-gene pairs (FDR = 30%) **(a)** and all *cis*-acting eQTL-gene pairs (FDR = 10%) **(b)** after empirical FDR correction between kruX (blue lower left set), parametric ANOVA (yellow upper set), and linear models (red lower right set).

Next we compared the output of kruX and matrix-eQTL’s parametric ANOVA and linear model (henceforth called "ANOVA" and "linear") methods. The Kruskal-Wallis test is more conservative than the ANOVA and linear methods, i.e. it has a higher nominal *P*-value for almost all marker-trait combinations (Figure 2). Since random data will be subjected to the same biases, nominal *P*-values cannot be directly compared to assess significance. We therefore performed empirical FDR correction for multiple testing using three randomly permuted datasets (cf. Implementation). Surprisingly, after FDR correction only a limited number of associations remained for ANOVA even at an FDR threshold of 30%, whereas the number of associations detected by kruX and the linear method was comparable (Figure 3(a)). Detailed analysis showed that this is due to pairing of SNPs with rare homozygous minor alleles (one or two samples) to genes with outlier expression levels, resulting in extremely low *P*-values for the ANOVA method in real as well as randomised data (see also below). To reduce the incidence of chance associations between singleton genotype groups and outlying expression values in the ANOVA method we repeated the empirical FDR correction, this time keeping only marker-trait combinations within 1Mbp of each other ("cis-eQTLs"). At an FDR threshold of 10% the number of significant *cis*-eQTL-gene pairs is indeed comparable between the three methods, with a large proportion of pairs detected by all three of them (Figure 3(b)).

### The Kruskal-Wallis test is more robust and detects more non-linear associations

**Figure 4:**
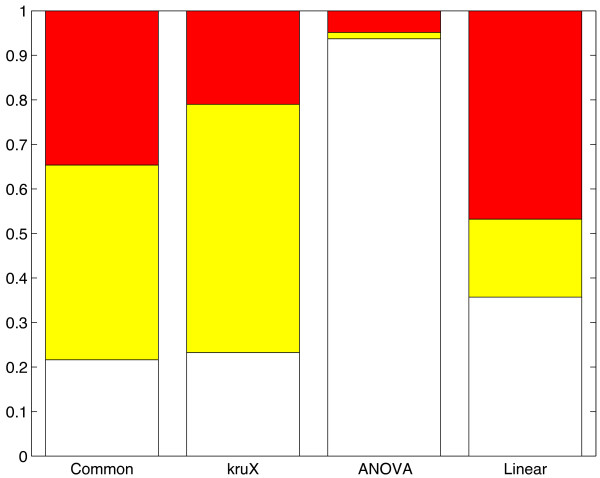
**Relative proportions of eQTL types.** Relative proportion of eQTL-types for cis-eQTLs common to all 3 methods and specific to each method; white (bottom), skewed genotype group sizes; yellow (middle), non-linear eQTLs; red (top), others. The absolute number of eQTLs in each group is 7,193 (Common), 1,663 (kruX), 701 (ANOVA) and 5,102 (Linear), cf. Figure 3(b).

**Figure 5:**
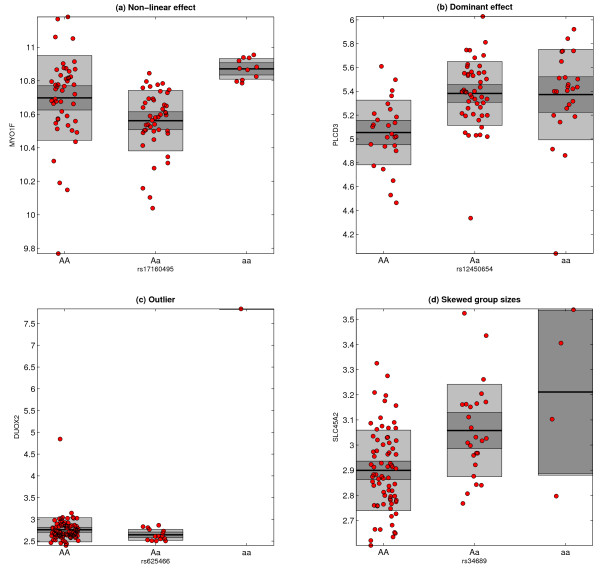
**Representative examples of eQTL associations.** **(a-b) Non-linear associations.** kruX identifies more non-linear relations where the gene expression level of the heterozygous samples lies outside the typical range of the homozygous samples **(a)** or where one allele has a dominant effect on the gene expression level **(b)**. **(c-d) Problematic associations.** Parametric ANOVA gives high significance to spurious associations for genes with outlying expression samples that coincide with singleton genotype groups **(c)**. Associations with skewed genotype group sizes where the model assumptions are difficult to ascertain achieve high significance using linear models **(d)**.

We classified eQTL-gene pairs as "skewed group sizes" (smallest genotype group less than 5 elements), non-skewed "non-linear" [median of heterozygous and homozygous samples significantly different (Wilcoxon rank sum *P* < 0.05)] and non-skewed "other" (all others). *Cis*-associations identified exclusively by the Kruskal-Wallis test are more often non-linear and the overall distribution of eQTL-types is more similar to associations identified by all three methods, compared to the ANOVA and linear methods (Figure 4 and Figure 5(a-b)). Of the 701 associations exclusively identified using the parametric ANOVA method, 657 (94%) had skewed group sizes, including 426 (61%) with a singleton genotype group (the aforementioned ‘outliers’, cf. Figure 5(c)). The associations exclusively identified by the linear method also contained a much higher proportion of SNPs with skewed group sizes than the corresponding kruX associations (36% vs. 23%) and, as expected, a reduced number of non-linear associations (Figure 4 and Figure 5(d)).

## Conclusions

We have developed kruX, a software tool that uses matrix multiplications to simultaneously calculate the Kruskal-Wallis test statistics for millions of marker-trait combinations in a single operation, thereby realising a dramatic speed-up compared to calculating the test statistics one-by-one. The availability of a fast method to identify eQTL associations using a non-parametric test allowed us to assess in more detail how differences in model assumptions compared to parametric methods lead to differences in identified eQTLs. Our results on a typical human dataset indicate that the the parametric ANOVA method is highly sensitive to the presence of outlying gene expression values and SNPs with singleton genotype groups. We caution against its use without prior filtering of such outliers. Linear models reported the highest number of eQTL associations after empirical FDR correction. These are understandably biased towards additive linear associations and were also sensitive to the presence of skewed genotype group sizes, albeit to a much lesser extent than the parametric ANOVA method. The Kruskal-Wallis test on the other hand is robust against data outliers and heterogeneous genotype group sizes and detects a higher proportion of non-linear associations, but it is more conservative for calling additive linear associations than linear models, even after FDR correction.

In summary, kruX enables the use of non-parametric methods for massive eQTL mapping without the need for a high-performance computing infrastructure.

## Availability and requirements

• **Project name:** kruX

• **Project home page:**http://krux.googlecode.com

• **Operating systems:** Platform independent

• **Programming language:** Matlab, R, Python

• **Other requirements:** None

• **License:** GNU GPL v3

• **Any restrictions to use by non-academics:** None

## Competing interests

The authors declare that they have no competing interests.

## Authors’ contributions

JQ designed and implemented the algorithm and analysed the data; HFA analysed the data; JB provided validation data and co-supervised the project; TM designed and implemented the algorithm, analysed the data, supervised the project and drafted the manuscript. All authors read and approved the final manuscript.

## References

[B1] Hindorff LA Sethupathy P Junkins HA Ramos EM Mehta JP Collins FS Manolio TA Potential etiologic and functional implications of genome-wide association loci for human diseases and traits Proc Nat Acad Sci 2009 106 23 9362 9367 10.1073/pnas.0903103106 19474294 PMC2687147

[B2] Schadt EE Molecular networks as sensors and drivers of common human diseases Nature 2009 461 218 223 10.1038/nature08454 19741703

[B3] Cookson W Liang L Abecasis G Moffatt M Lathrop M Mapping complex disease traits with global gene expression Nat Rev Genet 2009 10 3 184 194 10.1038/nrg2537 19223927 PMC4550035

[B4] Foss EJ Radulovic D Shaffer SA Ruderfer DM Bedalov A Goodlett DR Kruglyak L Gentic basis of proteome variation in yeast Nat Genet 2007 39 1369 1375 10.1038/ng.2007.22 17952072

[B5] Nicholson G Rantalainen M Li JV Maher AD Malmodin D Ahmadi KR Faber JH Barrett A Min JL Rayner NW A genome-wide metabolic QTL analysis in Europeans implicates two loci shaped by recent positive selection PLoS Genetics 2011 7 9 1002270 10.1371/journal.pgen.1002270 PMC3169529 21931564

[B6] Shabalin AA Matrix eQTL: ultra fast eQTL analysis via large matrix operations Bioinformatics 2012 28 10 1353 1358 10.1093/bioinformatics/bts163 22492648 PMC3348564

[B7] Kruglyak L Lander ES A nonparametric approach for mapping quantitative trait loci Genetics 1995 139 3 1421 1428 7768449 10.1093/genetics/139.3.1421 PMC1206467

[B8] Schadt EE Molony C Chudin E Hao K Yang X Lum PY Kasarskis A Zhang B Wang S Suver C Zhu J Millstein J Sieberts S Lamb J GuhaThakurta D Derry J Storey JD Avila-Campillo I Kruger MJ Johnson JM Rohl CA van Nas A Mehrabian M Drake TA Lusis AJ Smith RC Guengerich FP Strom SC Schuetz E Mapping the genetic architecture of gene expression in human liver PLoS Biol 2088 6 107 10.1371/journal.pbio.0060107 PMC2365981 18462017

[B9] Kruskal WH Wallis WA Use of ranks in one-criterion variance analysis J Am Stat Assoc 1952 47 260 583 621 10.1080/01621459.1952.10483441

[B10] Leek JT Storey JD Capturing heterogeneity in gene expression studies by surrogate variable analysis PLoS Genet 2007 3 9 161 10.1371/journal.pgen.0030161 PMC1994707 17907809

[B11] Listgarten J Kadie C Schadt EE Heckerman D Correction for hidden confounders in the genetic analysis of gene expression Proc Nat Acad Sci 1646 107 38 5 16470 10.1073/pnas.1002425107 PMC2944732 20810919

[B12] Brem RB Kruglyak L The landscape of genetic complexity across 5,700 gene expression traits in yeast PNAS 2005 102 5 1572 1577 10.1073/pnas.0408709102 15659551 PMC547855

[B13] Hägg S Skogsberg J Lundström J Noori P Nilsson R Zhong H Maleki S Shang MM Brinne B Bradshaw M Bajic VB Samnegard A Silveira A Kaplan LM Gigante B Leander K de Faire U Rosfors S Lockowandt U Liska J Konrad P Takolander R Franco-Cereceda A Schadt EE Ivert T Hamsten A Tegner J Björkegren J Multi-organ expression profiling uncovers a gene module in coronary artery disease involving transendothelial migration of leukocytes and LIM domain binding 2: the Stockholm atherosclerosis gene expression (STAGE) study PLoS Genet 1000754 5 12 10.1371/journal.pgen.1000754 PMC2780352 19997623

